# Development and Validation of a Multidimensional Risk Spectrum Screening Tool for the Early Detection of Metabolic Syndrome

**DOI:** 10.7759/cureus.94932

**Published:** 2025-10-19

**Authors:** D A Gayanjalee, Shiwanthi Dharmapala, Ananda Chandrasekara

**Affiliations:** 1 Department of Nutrition and Dietetics, Wayamba University of Sri Lanka, Makandura, LKA

**Keywords:** cardiovascular disease, diabetes mellitus type 2, insulin resistance, metabolic changes and diabetes, metabolic syndrome (ms), risk assessment tools, south asian population, triglyceride-glucose index (tyg)

## Abstract

Background

Metabolic syndrome (MetS) is a cluster of risk factors, including dyslipidemia, hypertension, hyperglycemia, and central obesity, underpinned by insulin resistance (IR). Individuals with MetS have a markedly increased risk of developing type 2 diabetes mellitus and cardiovascular disease. Early detection before significant metabolic deterioration is essential for prevention.

Objective

The objective of this study is to develop and validate a practical screening tool for identifying individuals at varying risk levels for MetS by integrating anthropometric, biochemical, lifestyle, and demographic factors.

Methods

A literature review informed the selection of 23 modifiable and non-modifiable risk factors, including waist circumference (WC), BMI, fasting blood glucose (FBG), triglycerides (TG), high-density lipoprotein cholesterol (HDL-C), blood pressure (BP), lifestyle habits, and female-specific factors. Each factor was assigned a weighted risk score. The tool was pilot-tested and refined before being applied to 148 adults (47 (31.8%) men and 101 (68.2%) women; mean age, 42.4 ± 11.7 years) attending a clinical setting. Data were collected through structured interviews, anthropometric assessments, and biochemical tests. The triglyceride-glucose (TyG) index was calculated as a surrogate for IR. Differences in clinical parameters across risk categories were evaluated using one-way ANOVA.

Results

Participants were classified as low risk (31 (21.0%)), moderate risk (93 (63.0%)), or high risk (24 (16.2%)). MetS risk scores increased significantly with systolic BP, diastolic BP, WC, FBG, TG, and the TyG index, while HDL-C showed a significant negative association (all p < 0.01). The mean TyG index increased progressively from low (8.3 ± 0.4) to moderate (8.7 ± 0.4) to high risk (9.4 ± 0.3).

Conclusions

The MetS spectrum assessment tool demonstrated strong construct validity (through associations with established MetS diagnostic criteria) and criterion validity (via correlation with the TyG index), allowing early risk detection even in individuals who do not meet full diagnostic thresholds. Integration into clinical practice may facilitate timely intervention to prevent progression to MetS and related complications.

## Introduction

Metabolic syndrome (MetS) is characterized by a cluster of metabolic abnormalities that significantly increase the risk of non-communicable diseases (NCDs). Insulin resistance (IR), the underlying mechanism of MetS, drives progression toward type 2 diabetes mellitus and cardiovascular disease (CVD), with an estimated 30-40% progression rate within two decades [1,2]. MetS encompasses hyperglycemia, hypertension, visceral obesity, atherogenic dyslipidemia, endothelial dysfunction, and genetic susceptibility [3].

It is estimated that 20-25% of the world’s adult population has MetS, which is associated with approximately a twofold increase in all-cause mortality and a threefold increase in the risk of myocardial infarction or stroke compared with individuals without MetS [4]. According to WHO, 75% of non-pandemic-related deaths globally are due to NCDs. In 2021, 82% of premature NCD-related deaths and 73% of all NCD-related deaths occurred in low- and middle-income countries [5].

Recent meta-analyses indicate that South Asian populations exhibit significantly higher MetS prevalence rates (30-40%), largely attributable to rapid urbanization, lifestyle changes, and possible genetic predispositions [6]. These prevalence estimates, rather than incidence rates, are most relevant for developing screening tools in cross-sectional validation studies such as the present work.

Several diagnostic criteria have been proposed to define MetS, including those by the National Cholesterol Education Program Adult Treatment Panel III (NCEP-ATP III), the International Diabetes Federation (IDF), and WHO [7,8]. While these definitions share common elements, they differ in emphasis. The IDF and WHO criteria place greater focus on glucose metabolism and obesity, whereas the NCEP-ATP III definition emphasizes cardiovascular risk factors such as abdominal obesity, hypertriglyceridemia, elevated blood pressure (BP), low high-density lipoprotein cholesterol (HDL-C), and glucose intolerance [7,8].

According to the IDF, an individual is diagnosed with MetS if they meet the criteria for central obesity (waist circumference (WC) ≥90 cm in men and ≥80 cm in women for South Asians) plus at least two additional abnormalities: triglycerides (TG) ≥1.7 mmol/L (≥150 mg/dL), HDL-C <1.04 mmol/L (<40 mg/dL) in men or <1.3 mmol/L (<50 mg/dL) in women, systolic BP (SBP) ≥130 mmHg or diastolic BP (DBP) ≥85 mmHg, or fasting blood glucose (FBG) ≥5.6 mmol/L (≥100 mg/dL) [8]. In contrast, the NCEP-ATP III definition requires at least three of the following: abdominal obesity, elevated TG, low HDL-C, elevated BP, or impaired fasting glucose [7]. WHO definition emphasizes the presence of diabetes, impaired glucose tolerance, or IR combined with at least two additional factors: hypertension (≥140/90 mmHg or use of antihypertensive medication), dyslipidemia (TG ≥1.7 mmol/L and/or HDL-C <0.91 mmol/L in men or <1.01 mmol/L in women), obesity (BMI ≥30 kg/m² and/or waist-to-hip ratio >0.90 in men or >0.85 in women), or microalbuminuria (urinary albumin excretion ≥20 mg/min) [8].

These established definitions identify MetS only after significant metabolic changes have occurred, when IR is already present. Although the triglyceride-glucose (TyG) index has shown promise as a surrogate marker of IR, its routine use in primary care remains limited due to fragmented healthcare systems and lack of integration into screening protocols [9].

Furthermore, the established diagnostic frameworks primarily emphasize anthropometric and biochemical markers, often overlooking modifiable lifestyle behaviors that accelerate MetS development. These include unhealthy dietary patterns, physical inactivity, tobacco use, excessive alcohol consumption, poor sleep, and chronic stress [10,11]. Incorporating such lifestyle factors may enhance early detection. Notably, South Asians demonstrate increased metabolic risk at lower obesity thresholds (BMI ≥23 kg/m²; WC ≥90 cm in men and ≥80 cm in women) compared with Western populations [6].

The limitations of existing diagnostic approaches, particularly their inability to identify high-risk individuals before full syndrome manifestation, underscore the need for earlier detection strategies. Timely identification is especially important in South Asia, where MetS prevalence is high and preventive interventions can avert long-term complications. Since lifestyle interventions (such as dietary modification, increased physical activity, reduced alcohol and tobacco use, and stress management) effectively mitigate risk, a multidimensional tool that integrates anthropometric, biochemical, demographic, and lifestyle factors offers the potential to identify at-risk individuals earlier.

This study had two specific objectives: (1) to develop a multidimensional MetS spectrum assessment tool integrating anthropometric, biochemical, lifestyle, and demographic factors and (2) to validate the tool by assessing its construct and criterion validity against established MetS diagnostic parameters and the TyG index. These objectives aim to facilitate early detection of at-risk individuals in clinical practice.

## Materials and methods

Study design and participants

This was a cross-sectional validation study conducted among adults attending the Clinical Laboratory, Department of Nutrition and Dietetics, Wayamba University of Sri Lanka (Makandura, Sri Lanka). Data collection commenced on October 1, 2024 and concluded on October 30, 2024. A total of 148 participants (100%) were recruited, comprising 47 (31.8%) men and 101 (68.2%) women, with a mean age of 42.4 ± 11.7 years.

Inclusion criteria were adults aged ≥18 years without a prior diagnosis of diabetes or CVD. Pregnant or lactating women and individuals taking lipid-lowering or antihypertensive medications were excluded.

Development of the MetS spectrum assessment tool

A comprehensive literature review informed the selection of 23 modifiable and non-modifiable risk factors, including WC, BMI, FBG, TG, HDL-C, BP, lifestyle habits, and female-specific factors [12,13].

Physical activity and sleep domains were assessed using structured, interviewer-administered questionnaires adapted from the International Physical Activity Questionnaire and a validated sleep quality questionnaire, respectively [10,11]. The physical activity component captured the frequency and duration of moderate-to-vigorous activity over the preceding seven days, while the sleep assessment evaluated average daily sleep duration and quality during the same recall period. Each factor was assigned a weighted score based on its relative risk contribution.

Weight assignment for risk factors

The weighting of the 23 modifiable and non-modifiable risk factors was determined through a structured process that integrated empirical evidence and clinical relevance. An extensive review of epidemiological and clinical literature was conducted to identify the relative contribution of each factor to MetS risk and related cardiometabolic outcomes. Priority was given to risk factors consistently associated with IR, central obesity, dyslipidemia, hyperglycemia, and hypertension in large cohort studies and meta-analyses. The identified risk factors were then categorized into three levels based on their strength of association and clinical importance. High-impact risk factors such as WC, FBG, TG, HDL-C, and BP were assigned a weight of 2 points. Moderate-impact risk factors, including physical inactivity, dietary habits, sleep duration, stress, and family history, received a weight of 1 point. Demographic risk factors, such as age and sex, were assigned a weight of 0-1 point depending on established relative risk.

This tiered weighting approach reflected the differential contribution of each factor to overall metabolic risk. The final scoring matrix was reviewed by a panel of subject-matter experts in nutrition and metabolic disease to ensure clinical validity and ease of application in practice. The weighted scoring system was then applied during pilot testing to assess its feasibility, internal consistency, and discriminative ability before use in the main study.

Pilot testing and psychometric evaluation

The preliminary version of the MetS spectrum assessment tool was pilot-tested among 10 adults recruited from the same clinical setting. Each assessment was conducted by trained research assistants, with an average completion time of approximately 30 minutes per participant. Inter-rater reliability was evaluated using Cohen’s κ, which indicated good agreement across raters (κ = 0.82). Internal consistency, assessed using Cronbach’s α, demonstrated acceptable reliability for the overall scale (α = 0.84). Item-total correlations ranged from 0.42 to 0.73, indicating adequate item discrimination. Receiver operating characteristic analysis yielded an area under the curve of 0.86 (95% CI: 0.78-0.93), supporting the tool’s predictive validity for identifying individuals at elevated metabolic risk. Minor wording refinements were made to enhance clarity and ease of administration prior to field deployment. The complete instrument (item text, scoring, and cutoffs) is provided in Appendix A.

Data collection and measurements

Anthropometric parameters (weight, height, and WC) were measured according to WHO protocols. BMI was calculated as weight in kilograms divided by height in meters squared (kg/m²). BP was measured twice in a seated position using a calibrated sphygmomanometer, and mean values of SBP and DBP were recorded. Fasting venous blood samples were collected for FBG, TG, and HDL-C analysis using standardized enzymatic assays.

The TyG index was calculated using the formula:

$\text{TyG index} = \ln \left( \frac{\text{Triglycerides (mg/dL)} \times \text{Fasting Blood Glucose (mg/dL)}}{2} \right)$

This index has been validated as a surrogate marker for IR [9].

Sample size and statistical power

Sample size determination was based on two analytical objectives: (1) to assess the correlation (construct validity) between the MetS spectrum score and the TyG index, and (2) to examine differences in metabolic parameters across risk groups (known-groups validity).

Correlation/Construct Validity

A moderate Pearson correlation of r = 0.30 was assumed, based on previous studies [9,14]. Using Fisher’s z-transformation, the required sample size (n) was calculated as:

$n = \frac{(Z_{1-\alpha/2} + Z_{1-\beta})^2}{\left[ 0.5 \cdot \ln\left( \frac{1 + r}{1 - r} \right) \right]^2} + 3,$

where

\(Z_{1-\alpha/2} = 1.96 \quad \text{(two-sided } \alpha = 0.05), \quad Z_{1-\beta} = 0.84 \quad \text{(power = 0.80)}, \quad r = 0.30.\)

Hence, a minimum of 85 participants was required to achieve sufficient power to detect the assumed correlation.

Known-Groups/ANOVA Validity

To detect differences in metabolic markers across three risk categories (low, moderate, and high), a one-way ANOVA was planned. For a medium effect size (Cohen’s f = 0.25), α = 0.05, and power = 0.80, conventional sample size tables recommend approximately 159 participants [5]. Although our sample of 148 was slightly smaller, it still provided acceptable power to detect moderate-to-large effects (i.e., f ≥ 0.26).

For descriptive planning, South Asian prevalence estimates of MetS (~30-40%) were referenced to approximate the expected distribution across risk strata [6]. These prevalence data are most relevant to cross-sectional validation studies, whereas incidence rates are less applicable.

Statistical analysis

Data were analyzed using IBM SPSS Statistics for Windows, Version 26.0 (Released 2018; IBM Corp., Armonk, NY, USA). Descriptive statistics are presented as mean ± SD for continuous variables and n (%) for categorical variables. Between-group differences were assessed using one-way ANOVA for continuous variables and chi-square tests for categorical variables. Correlations were evaluated using Pearson’s coefficient. A two-sided p < 0.05 was considered statistically significant. Effect sizes (η² for ANOVA, r for correlations) and 95% CIs were reported where applicable [5,15].

Ethical considerations

Ethical approval for this study was obtained from the Ethics Review Committee of the Faculty of Livestock, Fisheries and Nutrition, Wayamba University of Sri Lanka (approval 202407H12). The study was conducted in accordance with the principles of the Declaration of Helsinki (2013 revision). Written informed consent was obtained from all participants prior to data collection. Participant anonymity and confidentiality were strictly maintained throughout the study.

## Results

Participant characteristics

A total of 148 participants (100%) were included in the analysis, comprising 47 men (31.8%) and 101 women (68.2%), with a mean age of 42.4 ± 11.7 years. Table 1 summarizes the baseline anthropometric and biochemical characteristics.

**Table 1:** Baseline anthropometric and biochemical characteristics of participants (n = 148) Data are presented as mean ± SD for continuous variables or n (%) for categorical variables. Between-group differences were assessed using independent t-tests (continuous variables) or chi-square tests (categorical variables). Two-sided significance was defined as p < 0.05. BP, blood pressure; DBP, diastolic blood pressure; FBG, fasting blood glucose; HDL-C, high-density lipoprotein cholesterol; SBP, systolic blood pressure; TG, triglycerides; WC, waist circumference.

Characteristic	n (%)	Mean ± SD
Age (years)		42.4 ± 11.7
≤40	65 (44.3)	32.2 ± 4.9
>40	83 (55.7)	50.5 ± 8.9
Gender		
Male	47 (31.8)	-
Female	101 (68.2)	-
BMI (kg/m²)		26.6 ± 4.5
Non-overweight (<23 kg/m²)	34 (23.0)	21.1 ± 1.7
Overweight/obese (≥23 kg/m²)	114 (77.0)	28.3 ± 3.7
FBG (mg/dL)		106.7 ± 26.4
Normal (<100)	63 (42.6)	92.5 ± 7.2
Elevated (≥100)	85 (57.4)	117.2 ± 30.3
Fasting TG (mg/dL)		131.9 ± 64.7
Normal (<150)	108 (73.0)	102 ± 28.2
Elevated (≥150)	40 (27.0)	212.5 ± 66.7
BP (mmHg)		SBP: 119.6 ± 17.3; DBP: 79.4 ± 10.9
SBP - Normal (≤120)	86 (58.1)	107.2 ± 7.7
SBP - Elevated (>120)	62 (41.9)	136 ± 12.8
DBP - Normal (≤80)	85 (57.4)	71.6 ± 6.6
DBP - Elevated (>80)	63 (43.0)	89.7 ± 5.7
WC (cm)		Male: 89.4 ± 8.1; female: 85.4 ± 11.4
Male - Normal (<90)	25 (53.2)	83.7 ± 5.1
Male - Elevated (≥90)	22 (46.8)	96.2 ± 5.2
Female - Normal (<80)	69 (29.7)	73 ± 6.6
Female - Elevated (≥80)	71 (70.3)	90.7 ± 8.5
HDL-C (mg/dL)		Male: 44.2 ± 15.8; female: 48.2 ± 9.1
Male - Normal (≥40)	33 (70.2)	49 ± 16.7
Male - Low (<40)	14 (29.8)	33.6 ± 5.1
Female - Normal (≥50)	32 (31.7)	59.1 ± 7.7
Female - Low (<50)	69 (68.3)	43.6 ± 4.1

Mean WC was significantly higher in men than in women (p < 0.05). Men also exhibited higher mean FBG, TG, and BP values, whereas women had higher mean HDL-C levels. These differences align with well-established gender-specific variations in metabolic risk profiles.

Behavioral and lifestyle characteristics

Behavioral and lifestyle risk factors are summarized in Table 2. Overall, 93 participants (63.0%) reported low levels of physical activity, while 38 participants (25.7%) reported frequent consumption of high-fat meals. Tobacco and alcohol use were more common among men (p < 0.05). Approximately one-third of participants reported sleep disturbances and high stress levels.

**Table 2:** Behavioral and lifestyle risk factors among participants (n = 148) Data are presented as n (%). Between-group differences were assessed using chi-square analysis.

Behavior	n (%)
Consumption of ≥2 servings of fruits per day	
>5 days per week	143 (96.6)
2-4 days per week	5 (3.4)
Rarely	0
Consumption of ≥3 servings of vegetables per day	
4 days or more	46 (31.1)
2-3 days	99 (66.9)
0-1 day	3 (2.0)
Weekly engagement in ≥30 minutes of physical activity	
4 days or more	53 (35.8)
2-3 days	40 (27.0)
0-1 day	55 (37.2)
Sleep duration	
≥7 hours	53 (35.8)
<6 hours	95 (64.2)
Smoking habit	
Never	144 (97.3)
Occasionally	2 (1.4)
Daily	2 (1.4)
Alcohol consumption	
Never	115 (77.8)
Monthly	28 (18.9)
Weekly	5 (3.4)
Daily	0

Distribution of MetS risk scores

**Figure 1:**
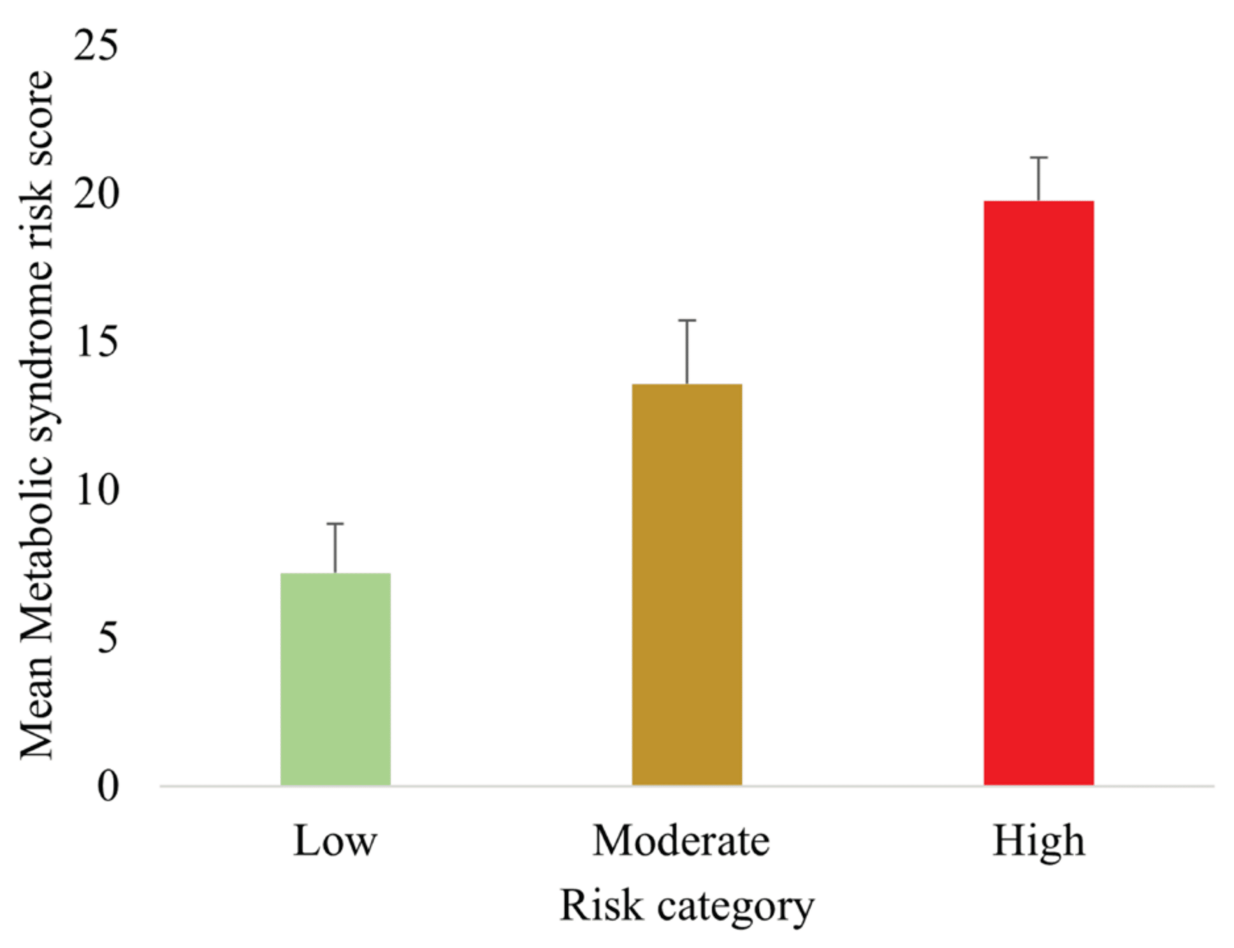
Mean MetS risk scores across low, moderate, and high categories Data are presented as mean ± SD (n = 148). Differences were assessed using one-way ANOVA with Tukey post hoc tests; all between-group comparisons were statistically significant (p < 0.05). MetS, metabolic syndrome

Using the newly developed MetS spectrum assessment tool, participants were stratified into three risk groups: low risk (31, 21.0%), moderate risk (93, 63.0%), and high risk (24, 16.2%). The distribution is presented in Table 3 and illustrated in Figure 1. The majority of participants fell into the moderate-risk group, highlighting the presence of subclinical metabolic risks in a large proportion of individuals who have not yet reached formal diagnostic thresholds.

**Table 3:** Distribution of participants across MetS spectrum risk categories (n = 148) Data are presented as n (%). MetS, metabolic syndrome

Risk category	MetS risk score	n (%)
Low risk	<9	31 (21.0)
Moderate risk	10-17	93 (63.0)
High risk	18-25	24 (16.2)
Very high risk	>26	0

BP across risk categories

**Figure 2:**
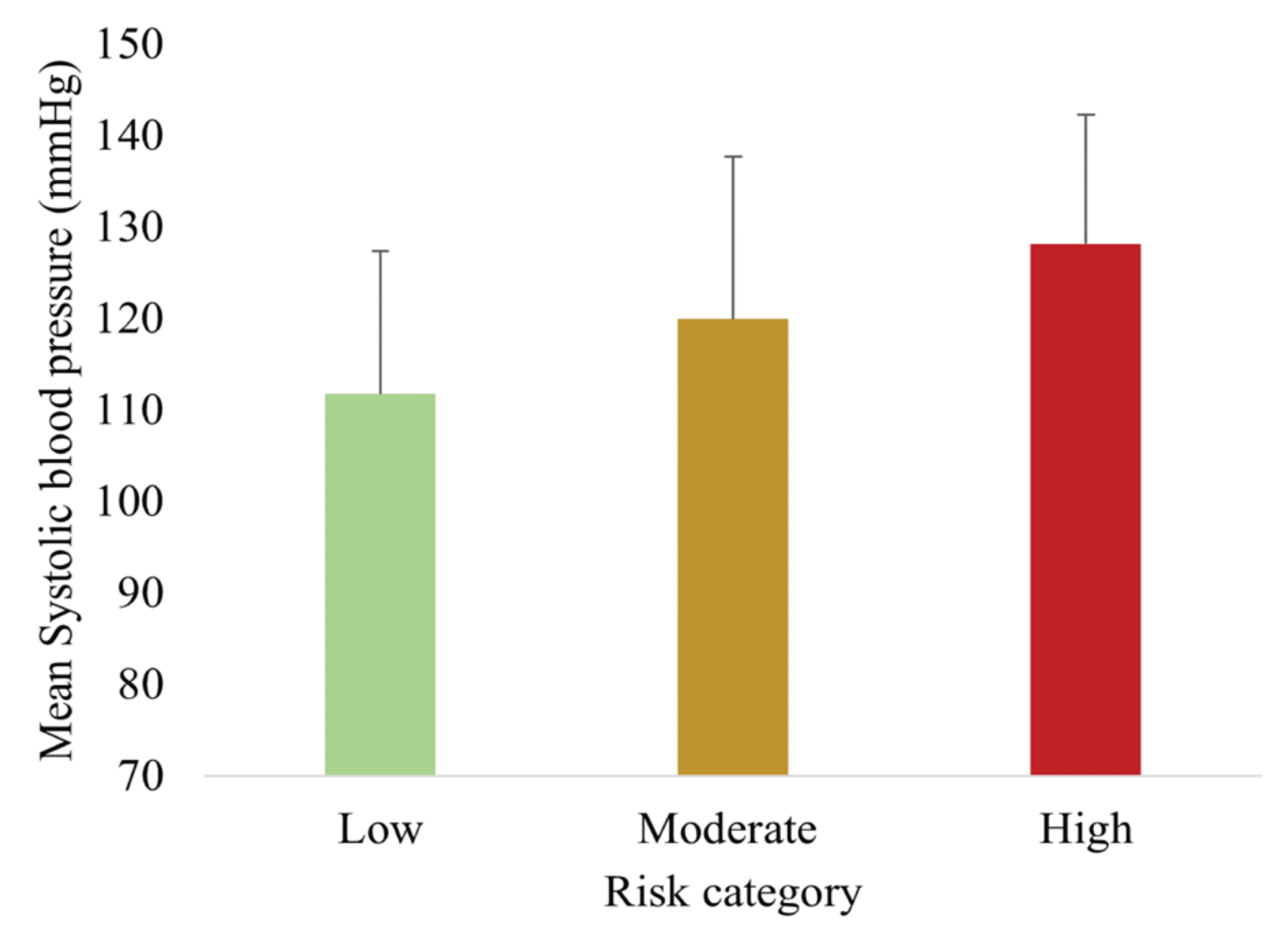
Mean SBP across low, moderate, and high MetS risk categories, demonstrating progressive elevation with higher risk Data are presented as mean ± SD (n = 148). Differences were assessed using one-way ANOVA with Tukey post hoc tests; all between-group comparisons were statistically significant (p < 0.05). Risk categories: Low <9, Moderate 10-17, High 18-25, and Very High >26 MetS, metabolic syndrome; SBP, systolic blood pressure

Figure 2 shows mean SBP across the three risk categories, progressively increasing from low to high risk (p < 0.05, ANOVA).

**Figure 3:**
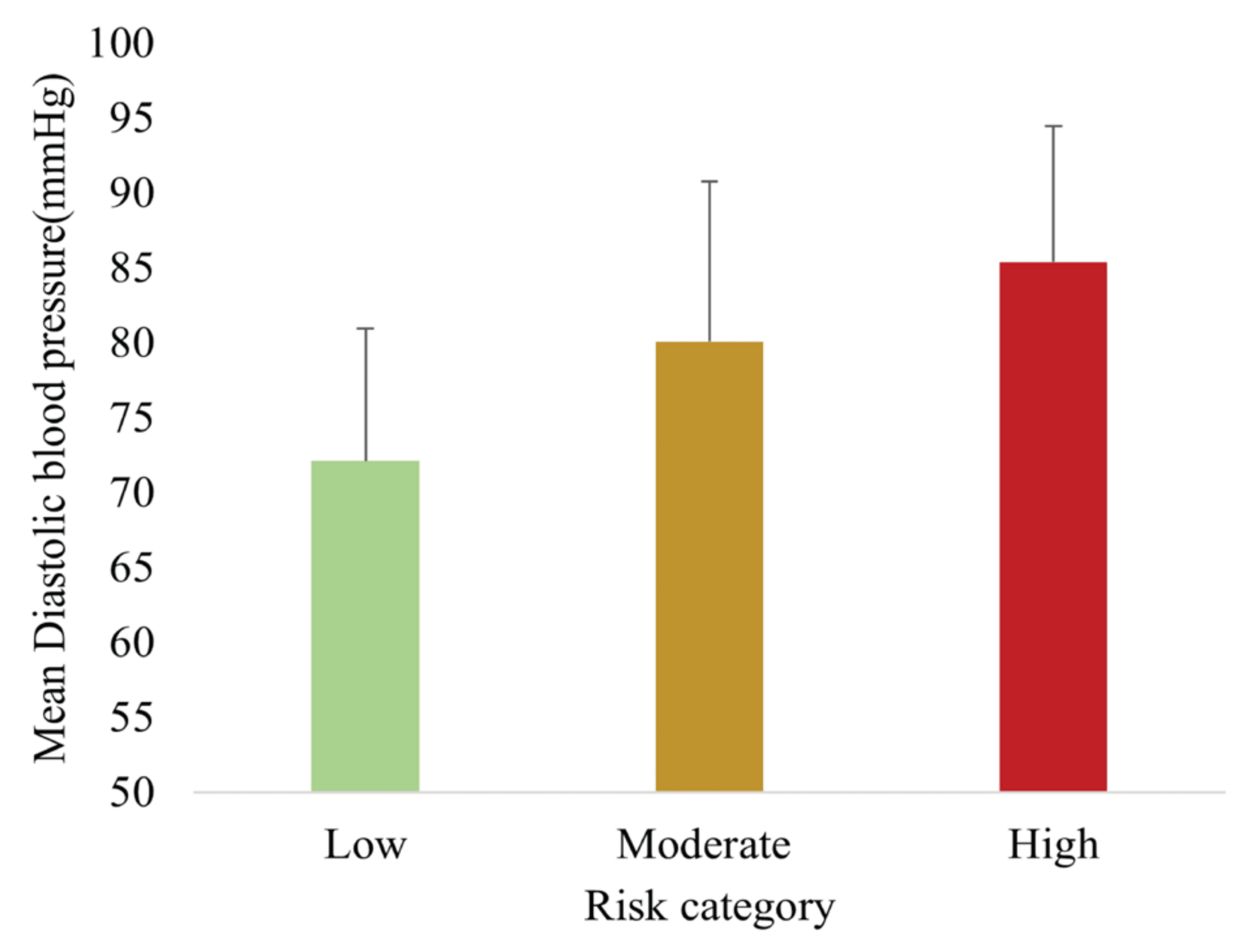
Mean DBP across low, moderate, and high MetS risk categories, showing progressive elevation with increasing risk Data are presented as mean ± SD (n = 148). Differences were assessed using one-way ANOVA with Tukey post hoc tests; all between-group comparisons were statistically significant (p < 0.05). DBP, diastolic blood pressure; MetS, metabolic syndrome

Figure 3 demonstrates that mean DBP also increased significantly with higher risk categories (p < 0.05). Together, these findings indicate that BP is a sensitive parameter distinguishing risk strata, even among participants not classified as hypertensive by conventional criteria.

Anthropometric measures across risk categories

**Figure 4:**
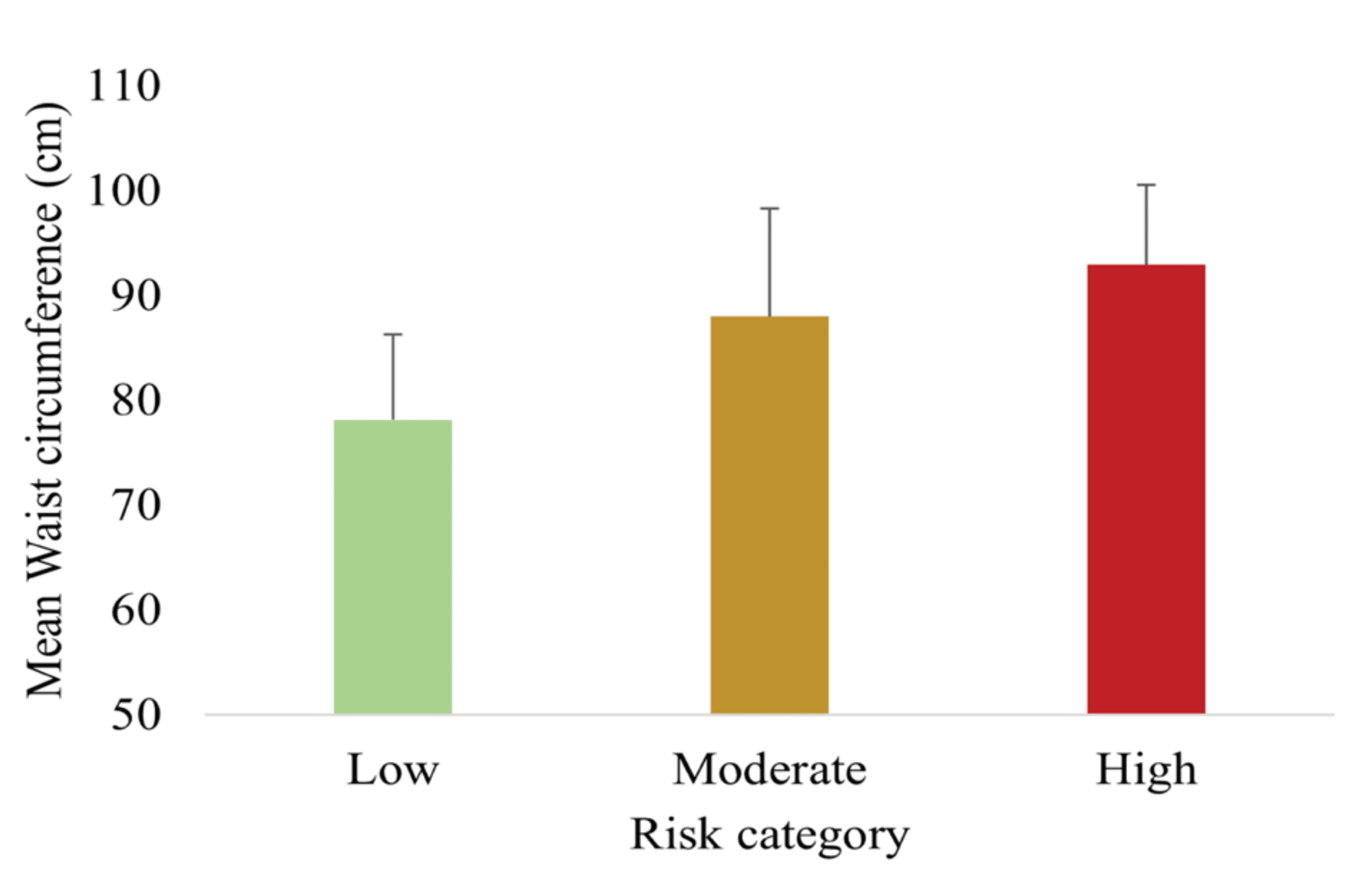
Mean WC across low, moderate, and high MetS risk categories, indicating increasing central obesity with higher risk Data are presented as mean ± SD (n = 148). Differences were assessed using one-way ANOVA with Tukey post hoc tests; all between-group comparisons were statistically significant (p < 0.05). MetS, metabolic syndrome; WC, waist circumference

Figure 4 depicts the mean WC across risk groups. WC increased significantly from low to high risk (p < 0.05), supporting central adiposity as a key discriminator of MetS risk in South Asian populations, consistent with established lower WC cutoffs for this population [6].

Biochemical measures across risk categories

**Figure 5:**
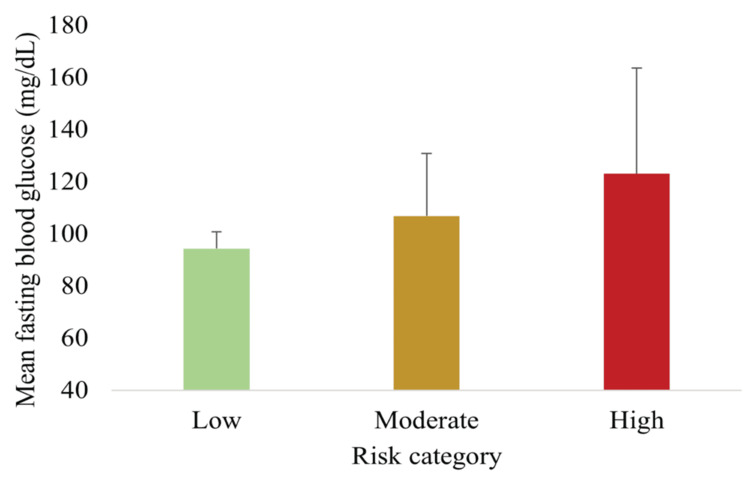
Mean FBG across MetS risk categories showing progressive increases across risk groups Data are presented as mean ± SD (n = 148). Differences were statistically significant (one-way ANOVA, p < 0.05). FBG, fasting blood glucose; MetS, metabolic syndrome

Figure 5 shows that the mean FBG increased progressively across risk categories (p < 0.05).

**Figure 6:**
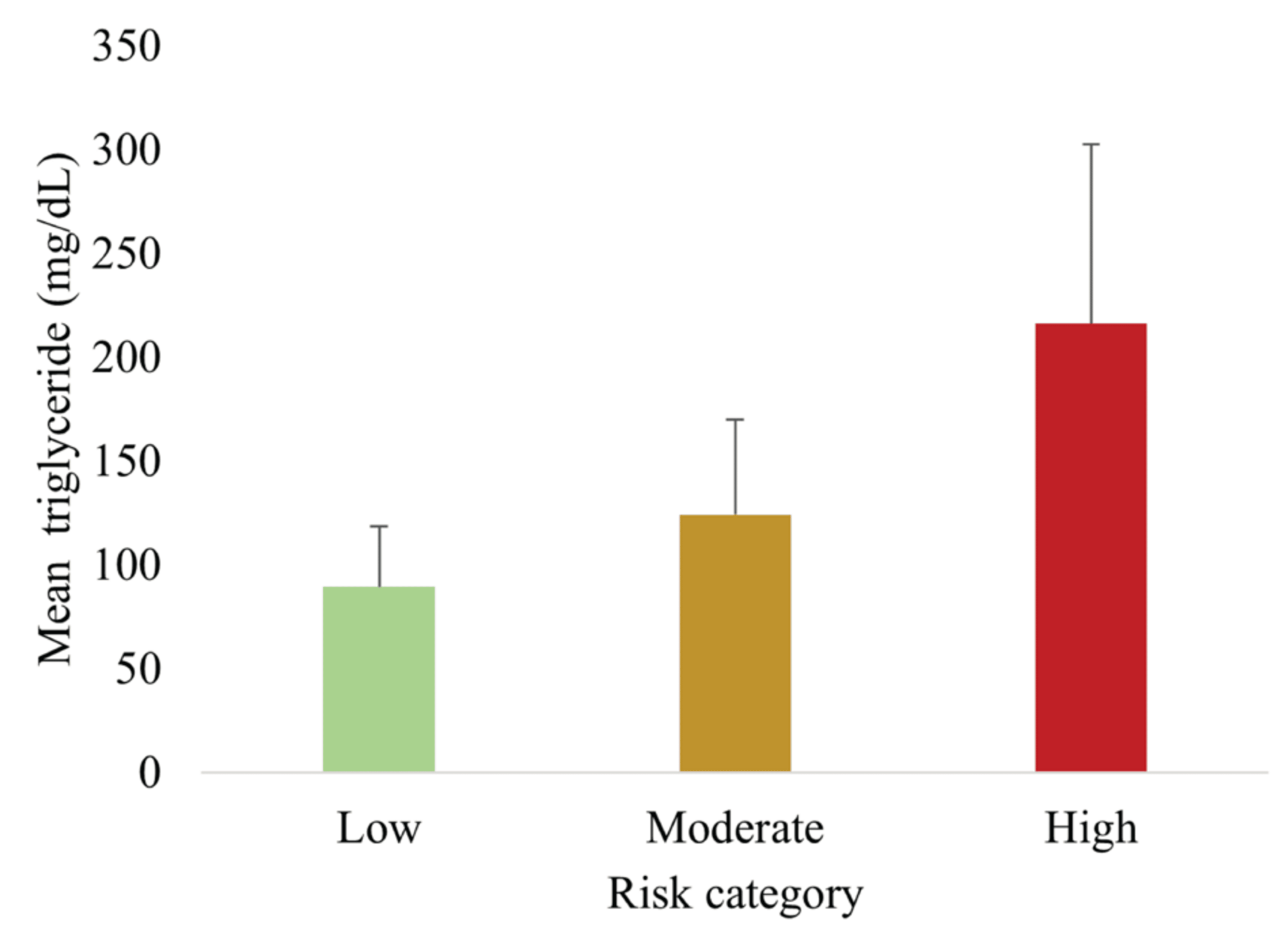
Mean TG levels across MetS risk categories showing a progressive increase across categories Data are presented as mean ± SD (n = 148). Significant between-group differences were observed (one-way ANOVA, p < 0.05). MetS, metabolic syndrome; TG, triglycerides

Figure 6 demonstrates that TG levels also rose progressively with higher risk scores (p < 0.05).

**Figure 7:**
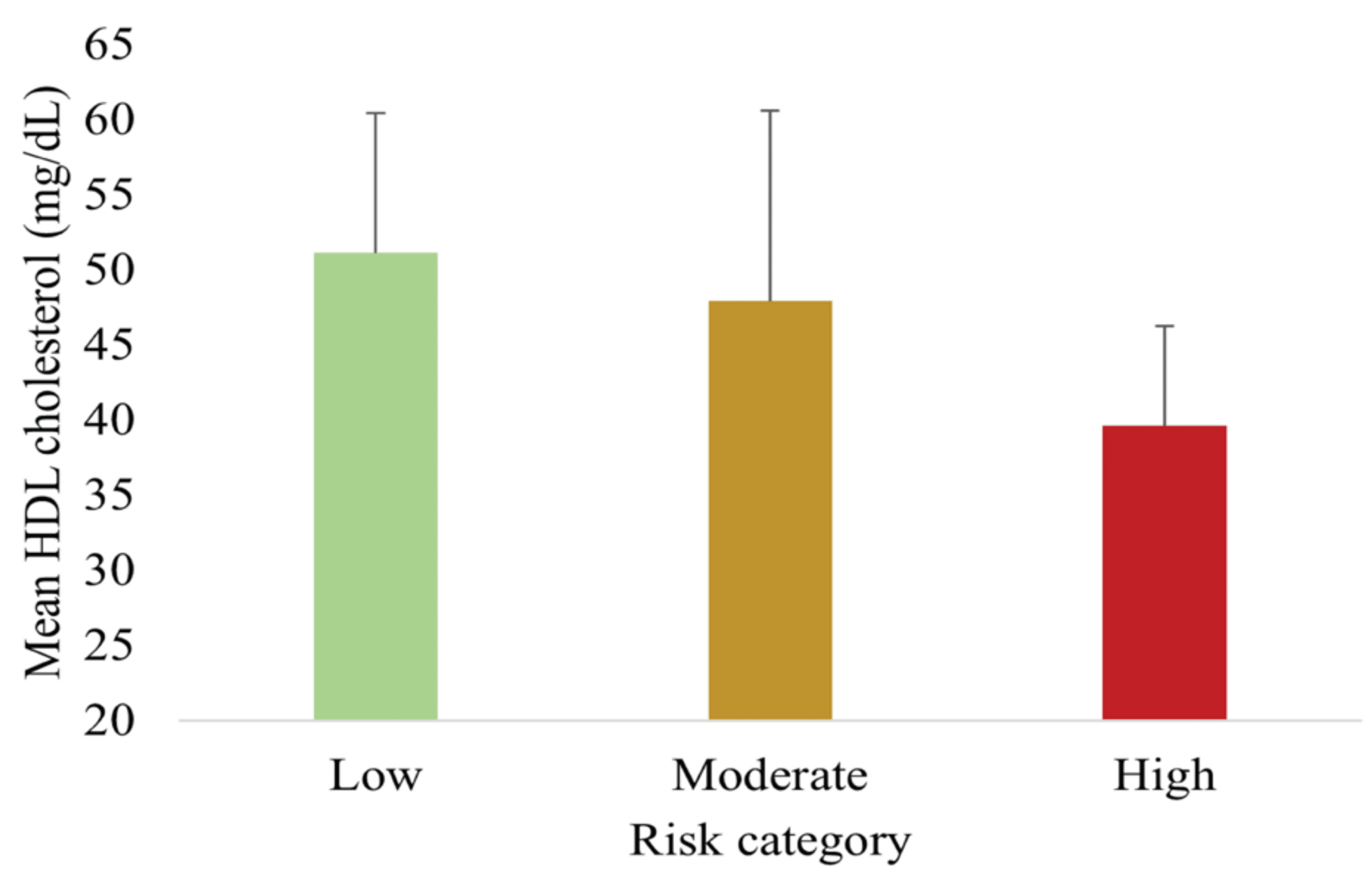
Mean HDL-C across MetS risk categories showing a significant decrease with increasing risk scores Data are presented as mean ± SD (n = 148). Differences were statistically significant (one-way ANOVA, p < 0.05). HDL-C, high-density lipoprotein cholesterol; MetS, metabolic syndrome

Conversely, Figure 7 shows that HDL-C decreased significantly with increasing risk (p < 0.05). These findings confirm that biochemical abnormalities correspond strongly with higher MetS spectrum scores, reinforcing the construct validity of the tool.

TyG index validation

**Figure 8:**
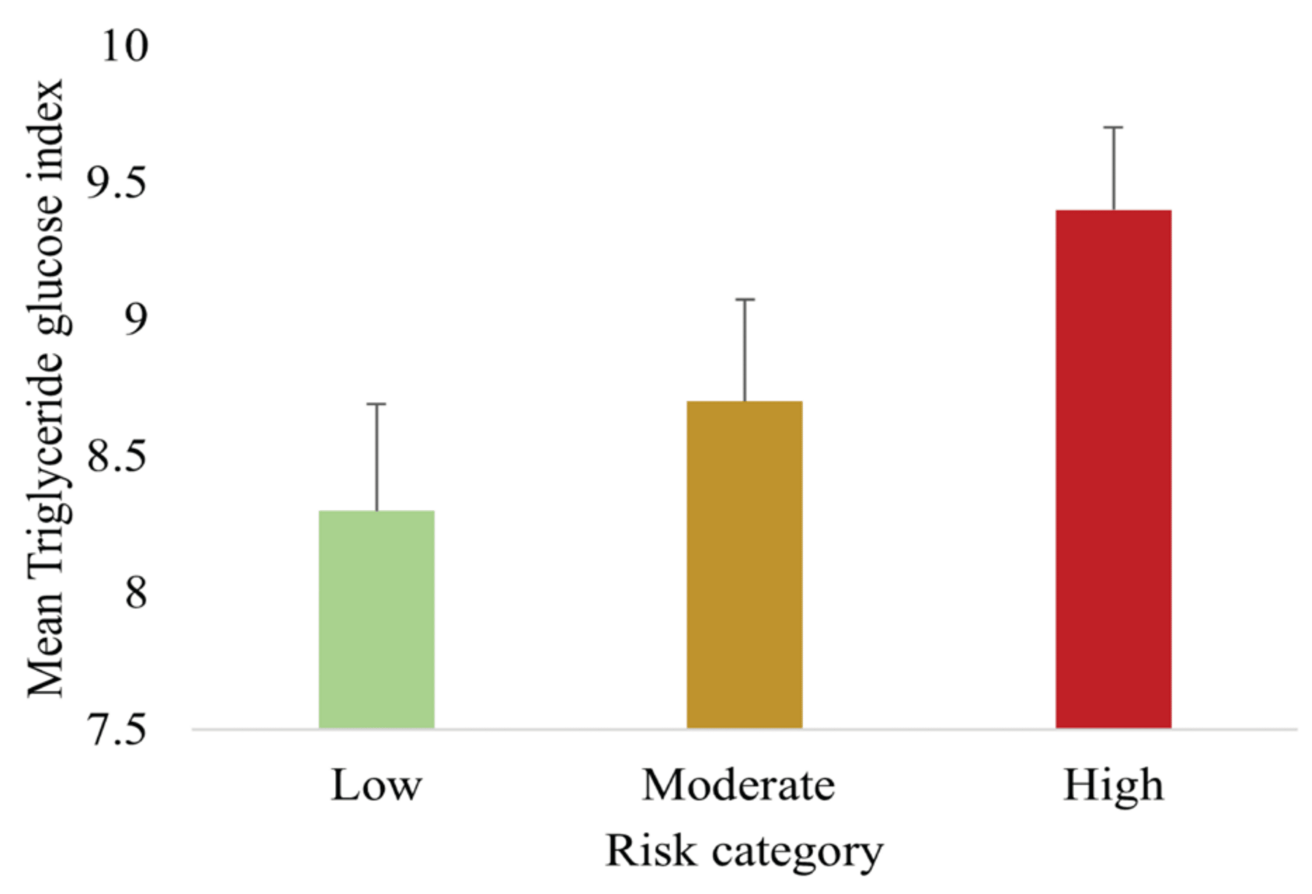
Mean TyG index across low, moderate, and high-risk categories of MetS showing a progressive increase in IR with higher risk classification Data are presented as mean ± SD (n = 148). Differences were assessed using one-way ANOVA with Tukey post hoc tests; all between-group comparisons were statistically significant (p < 0.05). IR, insulin resistance; MetS, metabolic syndrome; TyG, triglyceride-glucose

Figure 8 illustrates the distribution of the TyG index across risk groups. Mean TyG values increased significantly from low (8.3 ± 0.4) to moderate (8.7 ± 0.4) to high risk (9.4 ± 0.3) (p < 0.05, ANOVA). The graded rise in TyG across categories demonstrates strong criterion validity, as this index is widely recognized as a surrogate marker of IR [9].

The newly developed MetS spectrum assessment tool effectively stratified participants into low-, moderate-, and high-risk groups. The majority (63.0%) were classified as moderate risk, with 21.0% and 16.2% in the low- and high-risk groups, respectively. Anthropometric, biochemical, and lifestyle risk factors demonstrated clear trends across categories: SBP, DBP, WC, FBG, and TG increased significantly with higher risk scores, whereas HDL-C decreased. Lifestyle risk factors, including physical inactivity, high-fat dietary intake, alcohol use, and stress, were also more prevalent in higher-risk groups. Importantly, the TyG index rose progressively across risk categories, supporting both construct validity (association with established MetS parameters) and criterion validity (correlation with IR).

## Discussion

In the present study, we proposed an integrated approach to define the multidimensional MetS spectrum by developing and validating a new score that incorporates anthropometric, biochemical, demographic, and lifestyle components. Using this instrument, participants were categorized as low, moderate, or high risk, with the majority (63.0%) classified as moderate risk. This highlights that a considerable proportion of adults may already exhibit multiple metabolic risk factors, even in the absence of formal diagnostic criteria.

Comparison with existing diagnostic frameworks

Our findings align with established criteria from the National Cholesterol Education Program-Adult Treatment Panel III (NCEP-ATP III), the IDF, and WHO, which identify central obesity, dyslipidemia, glucose intolerance, and hypertension as core components of MetS. In this study, WC, FBG, TG, and BP increased progressively from low- to high-risk categories, while HDL-C decreased, supporting the compatibility of our tool with existing diagnostic frameworks.

Innovation of the MetS spectrum tool

Unlike traditional models that rely primarily on biochemical and anthropometric markers, our tool incorporates modifiable lifestyle factors such as physical inactivity, dietary habits, tobacco and alcohol use, and sleep quality. These behavioral determinants are increasingly recognized as critical drivers of IR and cardiometabolic risk. By integrating these domains, the tool allows for earlier and more comprehensive risk profiling.

Moreover, the TyG index, a validated surrogate marker of IR, showed a progressive increase with higher cardiometabolic risk, confirming both the construct validity (association with established MetS parameters) and criterion validity (correlation with an independent marker of IR) of the tool. This dual validation reinforces the robustness of the assessment system.

Relevance to South Asian populations

South Asians are at higher risk of developing MetS and its complications, with prevalence estimates of 30-40%, often at lower BMI and WC thresholds compared to Western populations. Our findings support the use of region-specific cutoff criteria, emphasizing the need for culturally adapted screening tools for early detection in this population.

Strengths and limitations

Key strengths of this study include the rigorous design, validation against traditional MetS criteria and the TyG index, and implementation in a South Asian clinical population. The use of structured questionnaires and standardized measurement techniques enhances reliability.

However, several limitations should be noted. First, the cross-sectional design precludes assessment of predictive validity; longitudinal studies are needed to determine whether risk categories predict incident diabetes or cardiovascular events. Second, while the sample size was adequate for correlation analyses, it was slightly small for detecting medium effect sizes in ANOVA. Third, the tool was validated in a single population; external validation in heterogeneous cohorts is necessary before broader application.

Implications and future directions

The MetS spectrum tool offers a simple, cost-effective screening approach for early risk identification in primary care and community settings, particularly in low- and middle-income countries where the burden of NCDs is high. The inclusion of lifestyle and behavioral domains provides a foundation for targeted preventive counseling and interventions.

Future research should evaluate the tool in larger, multicenter, and longitudinal cohorts to assess predictive validity. Refinement of scoring cutoffs may further improve calibration across diverse demographic subgroups.

## Conclusions

This study developed and validated a multidimensional MetS spectrum assessment tool that integrates anthropometric, biochemical, lifestyle, and demographic factors to generate a comprehensive risk profile. The tool demonstrated strong construct validity, reflected in graded differences across metabolic parameters, and criterion validity, confirmed by significant associations with biochemical markers and the TyG index. The tool enables personalized risk analysis by identifying high-risk individuals and their lifestyle habits. It can be applied seamlessly from clinical practice, such as consultations, to public health interventions for preventive care, providing a flexible framework for different aims and perspectives. The findings indicate that the tool effectively differentiates risk categories and shows strong correlations with established diagnostic markers, supporting both its construct and criterion validity. However, as this study employed a cross-sectional design, the results should be interpreted cautiously with regard to predictive capacity. Longitudinal validation studies are warranted to confirm the tool’s ability to predict future metabolic outcomes and progression to MetS.

## Appendices

Appendix A 

**Table 4:** Metabolic Syndrome Spectrum Assessment Tool (MS-SPECTRUM) The table lists all 23 assessment items, along with their corresponding response options or thresholds and the assigned score (0, 1, 2, or 3). Scores for each item are summed to calculate a total score for each participant. Based on the total score, participants are classified into one of four risk groups: Low risk (<9 points), Moderate risk (10-17 points), High risk (18-25 points), or Very high risk (>26 points). * PCOS, polycystic ovarian syndrome

Item	Response options/thresholds	Risk category contribution (0/1/2/3)	Item score
Age	≤40 years; >40 years	0; 1	
Gender	Female; Male	0; 1	
BMI (kg/m²)	<23; 23–24.9; ≥25	0; 1; 2	
Waist circumference	Men <90 cm; Men ≥90 cm; Women <80 cm; Women ≥80 cm	0; 1; 0; 1	
Fasting triglycerides	<150 mg/dL; ≥150 mg/dL	0; 2	
HDL cholesterol	Men ≥40 mg/dL; Men <40 mg/dL; Women ≥50 mg/dL; Women <50 mg/dL; On cholesterol medication	0; 2; 0; 2; 2	
Fasting glucose	<100 mg/dL; ≥100 mg/dL; On diabetes medication	0; 2; 2	
Blood pressure	Normal (≤120/≤80 mmHg); Elevated (>120/>80 mmHg); On hypertension medication	0; 2; 1	
History of CVD (MI/stroke)	No; Yes	0; 2	
Family history: Diabetes	No; Yes	0; 1	
Family history: CVD	No; Yes	0; 1	
Family history: Hypertension	No; Yes	0; 1	
Family history: Obesity	No; Yes	0; 1	
Family history: Dyslipidemia	No; Yes	0; 1	
Physical activity (≥30 min/week)	≥4 days; 2–3 days; 0–1 day	0; 1; 2	
Fruit consumption (2 servings/day)	>5 days/week; 2–4 days; Rarely	0; 1; 2	
Vegetable consumption (3 servings/day)	≥4 days; 2–3 days; 0–1 day	0; 1; 2	
Sleep duration	≥7 hours; <6 hours	0; 1	
Smoking	Never; Occasionally; Daily	0; 1; 2	
Alcohol intake	Never; Monthly; Weekly; Daily	0; 1; 2; 3	
Stress perception	Low; Moderate; High	0; 1; 2	
Female-specific: PCOS*	No; Yes	0; 1	
Female-specific: Menopause	No; Yes	0; 1	
Total of the item scores			
Risk category interpretation of total scores	Low risk <9		
	Moderate risk 10–17		
	High risk 18–25		
	Very high risk >26		
